# miRNA gene counts in chromosomes vary widely in a species and biogenesis of miRNA largely depends on transcription or post-transcriptional processing of coding genes

**DOI:** 10.3389/fgene.2014.00100

**Published:** 2014-04-29

**Authors:** Atanu Ghorai, Utpal Ghosh

**Affiliations:** Department of Biochemistry and Biophysics, University of KalyaniKalyani, India

**Keywords:** non-coding gene, miRNA, chromosome, intronic, intergenic, correlation coefficient, coding genes, disease-association

## Abstract

MicroRNAs target specific mRNA(s) to silence its expression and thereby regulate various cellular processes. We have investigated miRNA gene counts in chromosomes for 20 different species and observed wide variation. Certain chromosomes have extremely high number of miRNA gene compared with others in all the species. For example, high number of miRNA gene in X chromosome and the least or absence of miRNA gene in Y chromosome was observed in all species. To search the criteria governing such variation of miRNA gene counts in chromosomes, we have selected three parameters- length, number of non-coding and coding genes in a chromosome. We have calculated Pearson's correlation coefficient of miRNA gene counts with length, number of non-coding and coding genes in a chromosome for all 20 species. Major number of species showed that number of miRNA gene was not correlated with chromosome length. Eighty five percent of species under study showed strong positive correlation coefficient (*r* ≥ 0.5) between the numbers of miRNA gene vs. non-coding gene in chromosomes as expected because miRNA is a sub-set of non-coding genes. 55% species under study showed strong positive correlation coefficient (*r* ≥ 0.5) between numbers of miRNA gene vs. coding gene. We hypothesize biogenesis of miRNA largely depends on coding genes, an evolutionary conserved process. Chromosomes having higher number of miRNA genes will be most likely playing regulatory roles in several cellular processes including different disorders. In humans, cancer and cardiovascular disease associated miRNAs are mostly intergenic and located in Chromosome 19, X, 14, and 1.

## Introduction

MicroRNAs are the major key players to regulate the expression of coding genes (Lagos-Quintana et al., 2001). Genes are divided into two groups—coding genes that code proteins and non-coding genes that do not code functional proteins. More than 98% of human genome is constituted of non-coding DNA (Elgar and Vavouri, 2008). Recent data from the Encyclopedia of DNA Elements (ENCODE) project suggests that 80.4% of the DNA in human genome participates in “at least one bio-chemical RNA and/or chromatin associated event” (ENCODE Project Consortium, 2012) implicating the evidence of their regulatory functions. So, it has been established fact that non-coding genes can be transcribed to form non-coding RNAs that can play regulatory role over expression of coding genes. Such non-coding RNAs are found in all eukaryotes like fungi, plants, protozoans, and metazoans (Carrington and Ambros, 2003). miRNA is a sub-set of non-coding RNAs that are transcribed by RNA polymerase II to form primary miRNA (Pri-miRNA). Nuclear RNase III enzyme Drosha and its co-factor process pri-miRNA into 60 nt length precursor miRNA (pre-miRNA) (Chang and Mendell, 2007). The precursor miRNA has stem and loop structure and it is exported into cytoplasm by exportin 5 followed by cleavage with Dicer-TRBP complex to form mature miRNA (Zhang et al., 2007). This mature miRNA makes complex with Argonaute (Ago) proteins to form RNA induced silencing complex (RISC) (Nasser et al., 2008). The functional strand of miRNA stably associated with RISC and the other strand is called passenger strand. Now the mature miRNA guides the RISC complex to repress its targets, mainly at 3′-untranslated region (3′-UTR) of mRNA via short complementary sequence of 6–8 nucleotides (called “seed” sequence), inducing mRNA destabilization, degradation and/or inhibiting translation for protein synthesis (Kim, 2005; Nilsen, 2007; Filipowicz et al., 2008; Fabian et al., 2010). One miRNA can post-transcriptionally silence several 1000 of genes expressions and a single gene can be silenced by more than one miRNA too (Cui et al., 2006; Wouters et al., 2011). Tissue-specific expression of miRNA was also observed (Choudhury et al., 2013; Salvi et al., 2013).

miRNAs can be categorized into four sub-types such as intergenic (dme-mir-1, mmu-mir-7b, hsa-mir-7-2 etc.), intronic (dme-mir-2a-1, mmu-mir-199b, hsa-let-7d etc.), exonic (dme-let-7, mmu-mir-9-2, hsa-let-7a-2 etc.), and others (dme-mir-4949, mmu-mir-331, hsa-mir-632 etc.) depending on their genomic origin (Ying et al., 2010; Chien et al., 2011). miRNA precursors located in intronic and exonic region of protein coding gene are called intronic and exonic miRNA respectively. The miRNA precursors located in between two consecutive protein coding genes are termed as intergenic miRNA. The others category includes 3′UTR, 5′UTR and combinations of any two from intron, exon, 3′UTR and 5′UTR (Griffiths-Jones et al., 2008; Godnic et al., 2013). Various miRNAs are produced as per requirement of the cell but the regulation or detail mechanism(s) of biogenesis of miRNAs are yet to be elucidated. miRNA genes can have transcription start site (TSS) like the coding genes and transcription factors that are used for transcription of mRNA are also used for miRNA transcription (Aguda et al., 2008; Pichiorri et al., 2010; Wang et al., 2010a,b). Again, miRNA could also be produced in cell as a by-product of post-transcriptional processing of coding genes using splicing machinery and lariat de-branching enzymes bypassing the conventional nuclear miRNA biogenesis pathway by Drosha cleavage as mentioned above (Okamura et al., 2007; Flynt et al., 2010). These miRNAs are termed as “Mirtrons” (Berezikov et al., 2007; Chan and Slack, 2007; Ruby et al., 2007; Westholm and Lai, 2011; Havens et al., 2012).Therefore, it is not very clear whether biogenesis of miRNA is a separate, parallel process as that of coding gene expression or linked with it.

Numerous studies to date have established the role of miRNAs in diverse cellular processes like stem cell differentiation, heart development (Chen et al., 2006; Zhao et al., 2007; Ivey et al., 2008), insulin secretion (Plaisance et al., 2006), apoptosis (Lukiw and Pogue, 2007; Tarasov et al., 2007), aging (Kumamoto et al., 2008; Maes et al., 2008), immunity (Chen et al., 2004; Rodriguez et al., 2007), cell proliferation, metabolism (Hwang and Mendell, 2007; Taganov et al., 2007; Stefani and Slack, 2008) etc. Recent studies also revealed that certain miRNAs are strongly associated with various diseases such as diabetes (Ciccacci et al., 2013; van de Bunt et al., 2013), cancer (Calin and Croce, 2006; Blenkiron and Miska, 2007; Wuchty et al., 2012; Benetatos et al., 2013), cardiovascular disease (Zhao et al., 2005; Sayed et al., 2007; Zhao et al., 2007), neurodegenerative diseases (Hébert et al., 2008; Wang et al., 2008a,b) and hence miRNAs can be treated as potential biomarker or diagnostic tool (Keller et al., 2011; Fassina et al., 2012; Jones et al., 2012; Weiland et al., 2012; Cuk et al., 2013; Endo et al., 2013).

There is as such no report available regarding detailed chromosome-specific localization of miRNA genes or precursors in different species. Here we have extensively studied the miRNA gene counts in different chromosomes in various species including human and also collected the miRNAs associated with two diseases-cancer and cardiovascular in human only. We have calculated the correlation coefficient between miRNA gene counts with chromosome length, number of coding and non-coding genes in a chromosome and proposed the evolutionary conserved predominant way of biogenesis of miRNA.

## Results

### Variation of miRNA gene counts in the chromosomes of different species of metazoa

**Figure 1:**
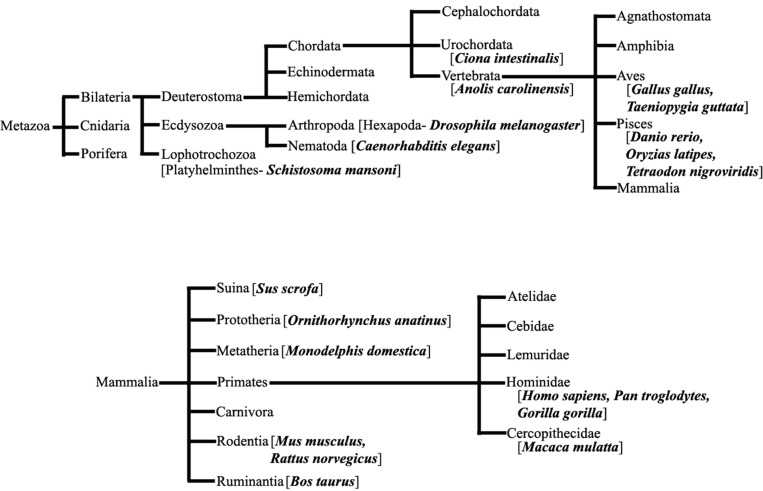
**Species under Metazoa.** The detail classification of 20 species (bold) which are chosen in this study.

**Figure 2:**
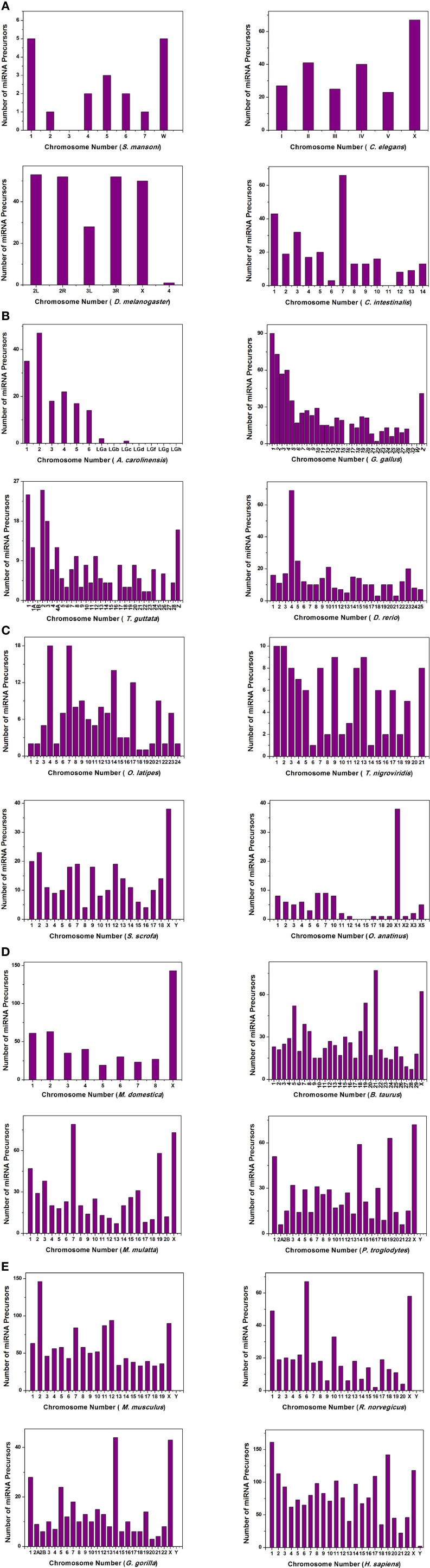
**The histogram represents the variation of miRNA precursors (from miRBase) in different chromosomes of species (A) *S. mansoni*, *C. elegans*, *D. melanogaster*, *C. intestinalis*; (B) *A. carolinensis*, *G. gallus*, *T. guttata*, *D. rerio*; (C) *O. latipes*, *T. nigroviridis*, *S. scrofa*, *O. anatinus*; (D) *M. domestica*, *B. taurus*, *M. mulatta*, *P. troglodytes*; (E) *M. musculus*, *R. norvegicus*, *G. gorilla*, *H. sapiens***.

We have extensively searched miRBase (Griffiths-Jones et al., 2008) to locate the miRNA precursors in different chromosomes and details of karyotype data are collected from Ensembl genome browser (Flicek et al., 2012) in 20 different species including human under metazoan. The detail classification of 20 species under study is shown in Figure 1. We have plotted number of miRNA precursors vs. chromosome number in all 20 species as shown in Figures 2A–E. It is evident from those figures that numbers of miRNA genes or precursors are widely varied throughout the all chromosomes of a particular species under study. Instead, there are certain chromosomes having higher counts of miRNA precursors ranging from *C. elegans* to *H. sapiens*. The number of miRNA precursors varies from 0 to 161 in a particular chromosome. For example, the highest number, i.e., 161 miRNA precursors are observed in Chromosome 1 whereas only 2 miRNA precursors are observed in Y chromosome in human. Chromosome 6 of *R. norvegicus* has the highest 67 miRNA precursors out of all the chromosomes but chromosome 16 has only 2 miRNA precursors in the same species. Importantly, the lowest or no miRNA precursors are observed in Y chromosomes, on the contrary high number of miRNAs is observed in X chromosomes in all the species. This observation suggests that presence of high number of miRNA genes in X chromosome and the least or absence of miRNA gene(s) in Y chromosome is an evolutionary conserved phenomenon. Different species have different number of chromosomes and the miRNA gene counts in chromosomes are non-uniform as evident from Figures 2A–E. Furthermore, we have selected four chromosomes of each species having top most number of miRNA genes as shown in Table 1 and calculated percentage of miRNA genes in the said four chromosomes. For example, Chromosome 1, 2, 4, and 3 are the top most miRNA genes containing chromosomes of *G. gallus* in decreasing order and as high as 39.66% miRNA genes come from these four chromosomes of this species; rest 30 chromosomes contribute ~60% miRNA genes.

**Table 1:** **Percentage of miRNA gene counts in top four highest number of miRNA gene containing chromosomes**.

**Sl no.**	**Species**	**Total no. of miRNA (A)**	**Ch no. (highest no. of miRNA) (B)**	**Ch no. (2nd highest no. of miRNA) (C)**	**Ch no. (3rd highest no. of miRNA) (D)**	**Ch no. (4th highest no. of miRNA) (E)**	**% miRNA contributed by top four highest miRNA gene containing Ch no. [(B+C+D+E)/A]×100 (%)**
1	*S. mansoni*	19	Ch W(5)	Ch 1(5)	Ch 5(3)	Ch 6(2)	78.94
2	*D. melanogaster*	236	Ch 2L(53)	Ch 2R(52)	Ch 3R(52)	Ch X(50)	87.71
3	*C. elegans*	223	Ch X(67)	Ch II(41)	Ch IV(40)	Ch I(27)	78.47
4	*C. intestinalis*	272	Ch 7(66)	Ch 1(43)	Ch 3(32)	Ch 5(20)	59.19
5	*A. carolinensis*	156	Ch 2(47)	Ch 1(35)	Ch 4(22)	Ch 3(18)	78.20
6	*G. gallus*	**706**	**Ch 1(90)**	**Ch 2(73)**	**Ch 4(60)**	**Ch 3(57)**	**39.66**
7	*T. guttata*	**225**	**Ch 2(25)**	**Ch 1(24)**	**Ch 3(18)**	**Ch Z(16)**	**36.88**
8	*D. rerio*	**347**	**Ch 4(69)**	**Ch 5(25)**	**Ch 10(21)**	**Ch 23(20)**	**38.90**
9	*O. latipes*	153	Ch 7(18)	Ch 4(18)	Ch 14(14)	Ch 17(12)	40.52
10	*T. nigroviridis*	113	Ch 2(10)	Ch 1(10)	Ch 9(9)	Ch 13(9)	33.62
11	*S. scrofa*	**266**	**Ch X(38)**	**Ch 2(23)**	**Ch 1(20)**	**Ch 12(19)**	**37.59**
12	*O. anatinus*	**106**	**Ch X1(38)**	**Ch 6(9)**	**Ch 7(9)**	**Ch 1(8)**	**60.37**
13	*M. domestica*	**441**	**Ch X(143)**	**Ch 2(63)**	**Ch 1(61)**	**Ch 4(40)**	**69.61**
14	*M. musculus*	**1183**	**Ch 2(146)**	**Ch 12(94)**	**Ch X(90)**	**Ch 11(87)**	**35.24**
15	*R. norvegicus*	**437**	**Ch 6(67)**	**Ch X(58)**	**Ch 1(49)**	**Ch 10(33)**	**47.36**
16	*B. taurus*	**801**	**Ch 21(77)**	**Ch X(62)**	**Ch 19(54)**	**Ch 5(52)**	**30.58**
17	*M. mulatta*	**558**	**Ch 7(79)**	**Ch X(73)**	**Ch 19(58)**	**Ch 1(47)**	**46.05**
18	*P. troglodytes*	**622**	**Ch X(72)**	**Ch 19(63)**	**Ch 14(59)**	**Ch 1(51)**	**39.38**
19	*G. gorilla*	327	Ch 14(44)	Ch X(43)	Ch 1(28)	Ch 5(24)	42.51
20	*H. sapiens*	**1876**	**Ch 1(161)**	**Ch19(142)**	**Ch X(118)**	**Ch 2(113)**	**28.46**

### Correlation of miRNA precursors with chromosomal length, number of coding/non-coding genes present in respective chromosome within a species

**Figure 3:**
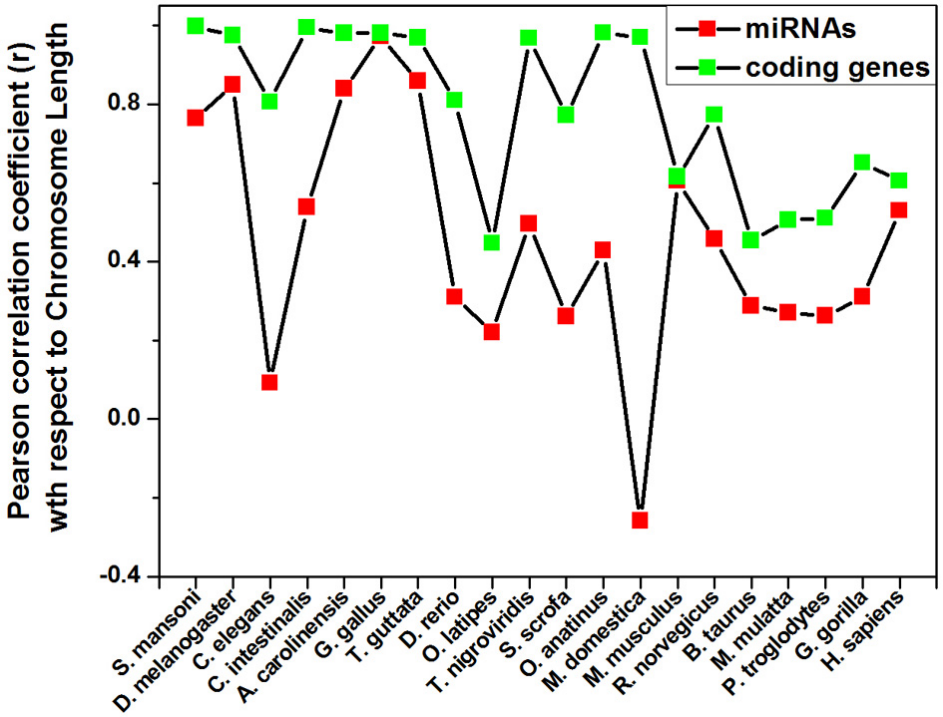
**Pattern of correlation coefficient of miRNAs and coding genes with chromosome length.** The Pearson correlation coefficient of miRNAs count and coding genes count with chromosome length of a species is calculated using Microsoft Office Excel 2003 software and plotted.

Different species have different number of chromosomes of variable length containing variable number of coding and non-coding genes. We have calculated the correlation coefficients of miRNA precursor counts with length of chromosome, number of coding/non-coding genes in all the 20 species as shown in Table 2. Majority of species (55%) under study showed that miRNA gene counts were not correlated with the chromosome length. So, longer chromosomes are not having higher number of miRNA precursors always. On the contrary, there are certain chromosomes in all species where higher number of miRNA genes located even if the length is shorter than rest of chromosomes and hence this is an evolutionary conserved event. For example, in *M. musculus* chromosome 1 (195.47 Mbps) is longer than chromosome 2 (182.11 Mbps) but the latter has the highest 146 miRNA genes whereas the earlier has 63 miRNA genes only. Then, we looked into the number of coding genes in different chromosomes. It has been observed that shorter chromosome can have more coding genes than longer one and maintained through evolution. The correlation coefficient (*r*-values, red square) of miRNA precursor counts with chromosomal length is just following the correlation coefficient (*r*-values, green square) of coding gene counts vs. chromosomal length in different species as shown in Figure 3. This data implicates that chromosomal localization of coding genes is someway related with that of miRNA genes in all the species under study.

**Table 2:** **Correlation coefficient of number of miRNA precursors vs. chromosome length, number of coding and non-coding genes in the respective chromosome**.

**Sl no.**	**Species**	**Correlation coefficient of number of miRNA precursors in respective chromosomes with**		
		**Chromosome length**	**Coding genes**	**Non-coding genes**
1	*S. mansoni*	0.76	0.77	0.62
2	*D. melanogaster*	0.85	0.85	0.75
3	*C. elegans*	0.09	−0.37	0.19
4	*C. intestinalis*	0.54	0.50	0.81
5	*A. carolinensis*	0.84	0.94	0.92
6	*G. gallus*	0.97	0.97	0.98
7	*T. guttata*	0.86	0.79	0.88
8	*D. rerio*	0.31	0.50	0.92
9	*O. latipes*	0.22	0.43	0.67
10	*T. nigroviridis*	0.50	0.47	0.63
11	*S. scrofa*	0.26	0.41	0.64
12	*O. anatinus*	0.43	0.46	0.78
13	*M. domestica*	−0.26	−0.15	−0.13
14	*M. musculus*	0.61	0.70	0.84
15	*R. norvegicus*	0.46	0.45	0.80
16	*B. taurus*	0.29	0.45	0.78
17	*M. mulatta*	0.27	0.55	0.66
18	*P. troglodytes*	0.26	0.55	0.50
19	*G. gorilla*	0.31	0.42	0.46
20	*H. sapiens*	0.53	0.91	0.78

Further, we calculated correlation coefficient of coding and non-coding genes with miRNA precursors present in a chromosome of all the species as shown in Table 2. The positive correlation has been noticed between miRNA vs. coding gene counts in all species under study except *C. elegans*. 55% (75% species if *r* ≥ 0.45) of species under consideration shows strong correlation coefficient (*r* ≥ 0.5) between number of miRNA genes and coding genes in a chromosome as evident from Table 2. Eighty five percent of species under consideration shows correlation coefficient (*r* ≥ 0.5) for miRNA vs. non-coding genes as expected because miRNA is sub-set of non-coding genes. We took four species showing *r*-value less than 0.5 and four species showing *r*-value greater than 0.5 and calculated percentage of each miRNA subtypes out of total miRNA precursors within the respective species as shown in Table 3. We have observed that *r*-value is greater than 0.5 when the sum of intronic and exonic miRNA precursors is greater than intergenic miRNA precursors in several species like *H. sapiens*, *T. guttata*, *A. carolinensis*, and *D. melanogaster*. On the contrary, *r*-value less than 0.5 is observed when number of intergenic miRNA precursors exceeds the total of intronic and exonic miRNA counts in species like *O. latipes*, *S. scrofa*, *O. anatinus*, and *G. gorilla* as shown in Table 3. These findings imply that intronic and exonic miRNA genes are closely related with coding genes in a chromosome and that too evolutionary conserved.

**Table 3:** **Number of miRNA sub-types in different species**.

**Species (*r*-value from Table 2)**	**Total miRNA precursors (under study)**	**Number of miRNA sub-types (%)**			
		**Intergenic %**	**Intronic %**	**Exonic %**	**Others %**
*H. sapiens* (0.87)	**1876**	**445 (23.72)**	**1059 (56.44)**	**110 (10.38)**	**262 (13.96)**
*T. guttata* (0.78)	242	56 (23.14)	34 (14.04)	101 (41.73)	51 (21.07)
*A. carolinensis* (0.93)	261	88 (33.71)	64 (24.52)	71 (27.20)	38 (14.55)
*D. melanogaster* (0.85)	236	75 (31.77)	115 (48.72)	21 (8.89)	25 (10.59)
*O. latipes* (0.42)	164	92 (56.09)	21 (12.80)	41 (25.00)	10 (6.09)
*S. scrofa* (0.38)	227	109 (48.01)	53 (23.34)	39 (17.18)	26 (11.45)
*O. anatinus* (0.35)	396	251 (63.38)	106 (26.76)	23 (5.80)	16 (4.04)
*G. gorilla* (0.41)	317	166 (52.36)	89 (28.07)	29 (9.14)	33 (10.41)

### Diversity of miRNA sub-types in human chromosomes

**Figure 4:**
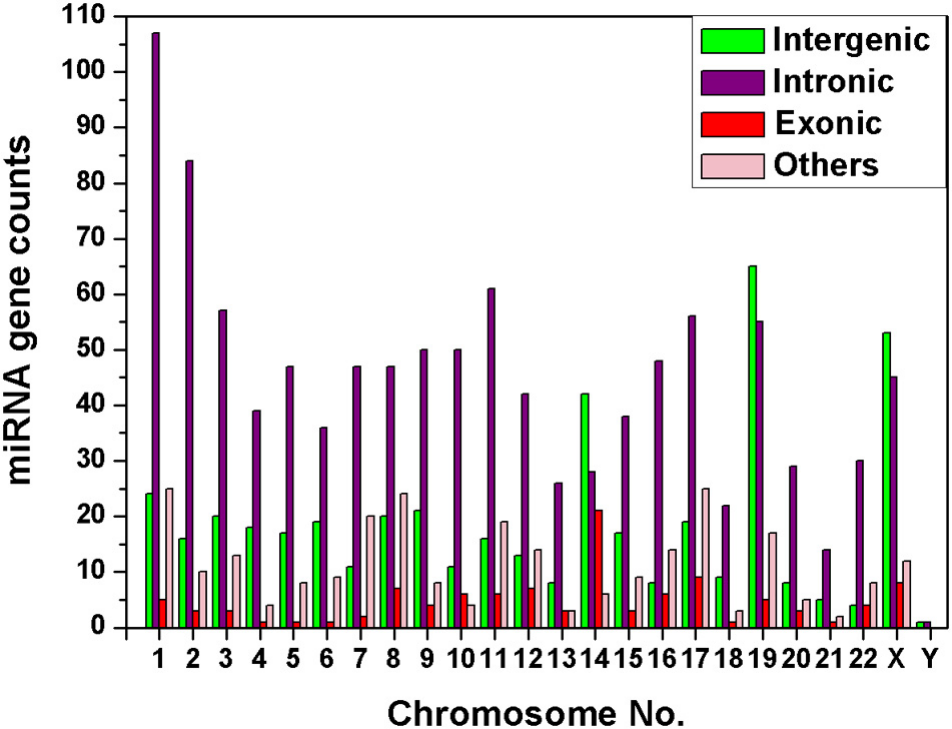
**Diversity of miRNA sub-types.** The histogram depicts the variation of number of miRNA sub-types—intergenic, intronic, exonic, and others in each of the chromosomes of human genome.

We have observed that miRNA genes are highly concentrated into certain chromosomes in all the 20 species including human. We collected total 1876 miRNA precursors from miRBase (Griffiths-Jones et al., 2008) and categorized them into four groups according to their genomic location such as intergenic, intronic, exonic and others. Chromosome wise variation of sub-types of miRNA gene counts are given in Figure 4. The four top most intergenic miRNA genes containing chromosomes are 19 (14.61%), X (11.91%), 14 (9.44%), and 1 (5.39%) whereas top most intronic miRNA genes containing chromosomes are 1 (10.10%), 2 (7.93%), 11 (5.76%), and 3 (5.38%). The exonic miRNA counts are very less than intergenic and intronic counts in a chromosome. We calculated the correlation of miRNA sub-types with coding genes. Overall correlation coefficients of coding genes vs. intergenic, intronic, exonic and others miRNAs are given by 0.47, 0.91, 0.25, and 0.74 respectively, indicating that intronic and others (3′UTR/5′UTR or combination) miRNA precursors are strongly correlated with coding genes in human.

### miRNAs associated with cancer and cardiovascular diseases in human chromosomes

**Figure 5:**
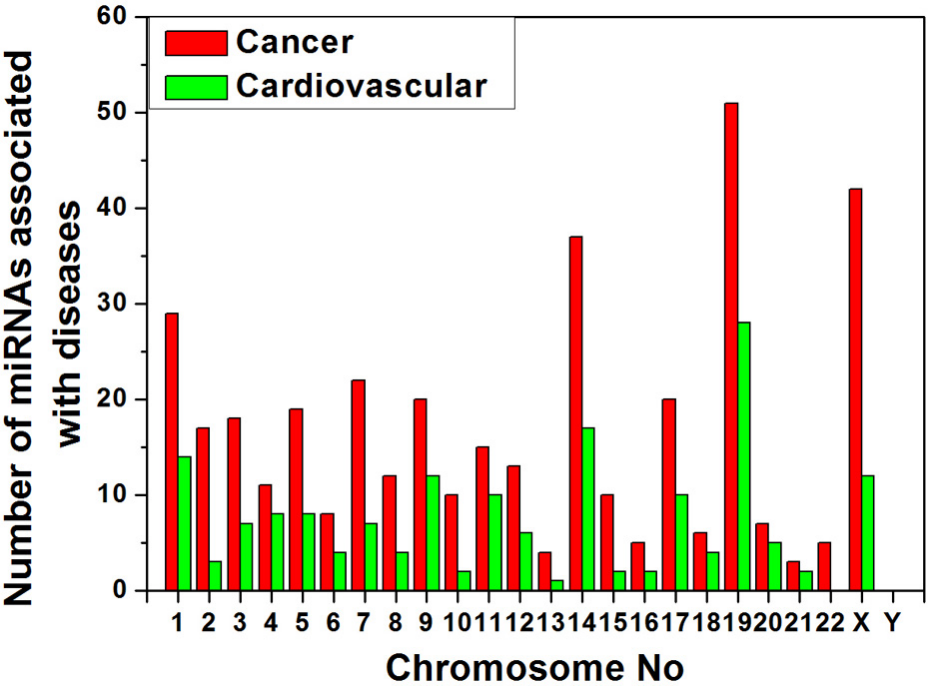
**Variation of number of miRNAs associated with cancer and cardiovascular disease in human chromosomes**.

We collected data of miRNA- disease association from HMDD v1.0 (January, 2012) which includes 617 miRNAs (Lu et al., 2008). We have categorized the miRNAs that are associated with cancer and cardiovascular disease separately and searched their location in chromosomes from miRBase database (Griffiths-Jones et al., 2008). The variation of counts of miRNA gene associated to the diseases in different human chromosomes is shown in Figure 5. We have observed that the counts of disease related miRNA gene widely varied into all chromosomes as expected but concentrated in certain chromosomes. Chromosomes 19, X, 14, 1 are the major cancer-associated miRNA gene containing chromosomes whereas chromosome 19, 14, 1, X are the major cardiovascular disease-associated miRNA gene containing chromosomes in decreasing order. Therefore, all the four chromosomes 19, 14, 1, and X are the major source of these two disease associated miRNA genes.

**Figure 6:**
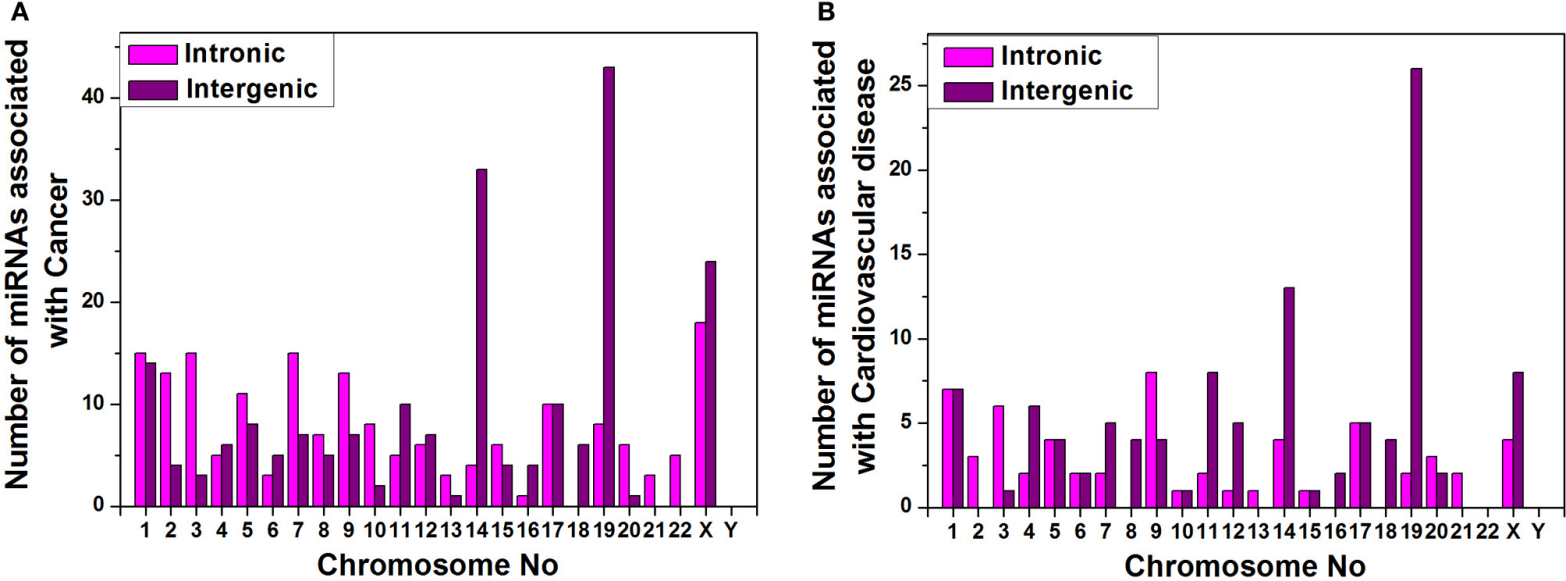
**miRNA sub-types associated with diseases.** Counts of intronic and intergenic miRNAs associated with cancer **(A)** and cardiovascular disease **(B)** in different human chromosomes.

Furthermore, we have arranged the miRNAs associated with cancer and cardiovascular disease according to their genomic origin like intronic or intergenic using miRBase database resources and plotted number of miRNA sub-types associated with cancer (Figure 6A) or cardiovascular disease (Figure 6B) vs. chromosome number. Bar diagram of Figures 6A,B shows that disease-associated intronic or intergenic miRNA gene counts are different in different chromosomes. For example, maximum intergenic miRNAs associated with both cancer and cardiovascular diseases are from Chromosome 19 followed by chromosome 14 and X as shown in Figures 6A,B. On the contrary, the highest number of intronic miRNAs associated with cancer and cardiovascular diseases are from chromosome X and 9 respectively.

We calculated the percentages of miRNA sub-types (intergenic and intronic) associated with cancer and cardiovascular disease out of total miRNA precursors of that particular sub-type (Table 4). We see that 45.84% and 24.26% intergenic miRNAs are associated with cancer and cardiovascular disease respectively. In a nutshell, higher proportions of inetrgenic miRNAs are associated with these two diseases compared with intronic sub-type and their major sources are chromosome 19, 14, and X.

**Table 4:** **Percentage of prevalence of miRNA sub-types associated with diseases**.

**Sub-type**	**Total counts of miRNA in human**	**Associated with cancer**		**Associated with cardiovascular disease**	
		**Total miRNAs**	**%**	**Total miRNAs**	**%**
Intronic	1059	180	16.99	60	5.66
Intergenic	445	204	45.84	108	24.26

## Discussion

We have investigated variation of miRNA gene counts in all the chromosomes of 20 different species. To investigate the criteria/logic behind such variation of miRNA gene counts in chromosomes, we have selected three parameters such as chromosomal length, number of coding and non-coding genes present in a chromosome and evaluated correlation coefficient of miRNA gene counts with length of chromosome, number of coding and non-coding genes. We have observed majority (55%) of species are not following the rule—“higher length chromosome contains higher number of miRNA genes.” So, length of chromosome is not only the criteria for chromosomal variation of miRNA gene counts. Surprisingly, there are certain chromosomes observed in all the species where the numbers of miRNA gene are extremely high. In human, Chromosome 1, 19, X, and 2 have high number of miRNA genes and constitute about 29% of total miRNA genes. Previously it was shown that miRNAs were non-randomly distributed in human genome and certain chromosomes had significantly higher number of miRNAs than other chromosomes (Calin et al., 2004). Similar study showed distribution of cancer-associated miRNAs in mouse genome (Sevignani et al., 2007). But, we have studied miRNA gene counts in chromosomes in 20 different species and observed certain chromosomes are having higher miRNA genes through evolution. For example, X chromosomes have high number of miRNA genes whereas Y chromosomes have least or no miRNA gene(s) in all the species. The reason behind such wide variation of miRNA gene counts in chromosomes is not clearly understood but this has come up as an evolutionary conserved phenomenon. It is reported that miRNAs are observed to be localized in chromosomes as clusters and co-expressed all the members from a single polycistronic transcript (Guo et al., 2014). The clustered miRNAs are expressed simultaneously and are involved in a particular pathway or a particular kind of cellular function. The number of miRNA genes in a cluster may be as high as 40 and such clustered pattern is maintained through evolution (Guo et al., 2014). The distribution of such clusters is again uneven and concentrated in to certain chromosomes in various species (Chan et al., 2012). For example, chromosome X and 14 has 40 and 31 number of clusters of miRNA in human (Chan et al., 2012). This data is supporting our observation that chromosome X and chromosome 14 have higher number of miRNA genes. Possibly, due to this clustering of miRNA we are getting high concentration of miRNA genes into certain chromosomes in various species.

Further, we evaluated Pearson's correlation coefficient between number of miRNA genes with number of coding and non-coding genes in a particular chromosome. About 85% species under study shows strong positive correlation (*r* ≥ 0.5) for non-coding genes. One obvious reason is that miRNA is a sub-set of non-coding RNA and a single primary transcript of non-coding RNA may produce more than one miRNA (Saini et al., 2007). We have observed strong positive correlation coefficient (*r* ≥ 0.5) between number of miRNA genes and coding genes in a chromosome as evident from Table 2. The relation between expressions of intronic, exonic and others miRNA with protein coding genes is very close. During the transcription of coding genes the introns left are used as source of intronic miRNAs. Intronic miRNAs or mirtrons can be spliced and debranched through Drosha-independent pathway to produce mature miRNA as observed in Drosophila (Flynt et al., 2010), nematodes (Ruby et al., 2007), avians (Glazov et al., 2008), mammals (Berezikov et al., 2007; Babiarz et al., 2008), and also plants (Zhu et al., 2008). Lots of reports showed co-expression of intronic miRNAs with corresponding host genes (protein coding genes) in various species, implicating that intronic miRNA and corresponding host protein coding genes are co-transcribed and both groups are under same regulatory elements such as promoter etc. (Lagos-Quintana et al., 2001; Lau et al., 2001; Rodriguez et al., 2004; Baskerville and Bartel, 2005; Chien et al., 2011). Therefore, array of mirtrons and host protein coding genes co-localize in chromosomes and we should get strong positive correlation between intronic miRNA-coding gene pair. We do get the correlation coefficient value *r* = 0.91 between intronic miRNA-mRNA pair in human. Earlier report showed strong positive (*r* > 0.5) Pearson's correlation coefficient of miRNA-mRNA pair in human using expression data (Wang and Li, 2009). There are reports where intronic miRNAs can also be transcribed using their own promoter regions and TSS (Corcoran et al., 2009; Wang et al., 2010a,b) although major portion of intronic miRNAs are co-transcribed with host protein coding genes. Similarly, exonic and others (includes 3′UTR, 5′ UTR and combinations of any two from intron/exon/3′UTR/5′UTR) miRNA are produced as a by-product of transcription and post-transcription processing of coding genes. It is to be noted that we do not check the expression of miRNAs or coding/non-coding genes but we have calculated correlation coefficient between miRNA-coding gene pair based on their chromosome-specific location. We have observed strong positive correlation (*r* > 0.5) when summation of intronic and exonic miRNA exceeds intergenic miRNA while *r* < 0.5 when intergenic exceeds the total of intronic and exonic miRNA genes in a particular species as evident from Table 3. miRNAs under others category are integral part of protein coding genes and can be expressed using transcription machinery of coding genes. Therefore, intronic, exonic and others miRNA genes co-localize with host protein coding genes through evolution and so there must be relation between expression of these miRNA genes with the corresponding coding genes. We hypothesize that intronic, exonic and others miRNAs are produced as by-product of transcription and post-transcriptional processing of corresponding host protein coding genes and this is an evolutionary conserved phenomenon.

Transcription of intergenic miRNAs and its relation with coding gene expression is poorly understood. Here, we have observed positive correlation (*r* = 0.47) between intergenic miRNA vs. coding genes in humans. The result indicates that expression of intergenic miRNA is less or no way related with expression of protein coding genes with compared to intronic miRNAs (discussed earlier). Previous studies showed that intergenic miRNAs can have its own promoters for transcription, independent of coding genes (Saini et al., 2007; Chien et al., 2011). But the promoters and TSS has similarity with that of coding genes (Corcoran et al., 2009). Sometimes more than one pre-miRNA may be produced from the same pri-miRNA transcript, forms cluster miRNAs (Altuvia et al., 2005), but how exactly intergenic miRNA transcription is related with that of coding gene is not known clearly. However, RNA polymerase II transcribes miRNA like mRNA and the same transcription factors are involved in miRNA transcription as that of mRNA transcription (Wang et al., 2010a,b).

The chromosomes having high number of miRNA genes are more crucial for several cellular processes including different diseases. We have observed that the miRNAs associated with cancer and cardiovascular disorders are concentrated into certain human chromosomes. An experimental observation showed that miRNAs were in cancer-associated genomic regions (CAGR) or in fragile sites (FRA) in different human chromosomes and certain chromosomes were found rich with cancer-associated miRNAs (Calin et al., 2004; Rossi et al., 2008). A statistically significant association between chromosomal location of miRNAs and tumor susceptibility loci was reported in mouse model and such miRNAs were unevenly distributed in the mouse chromosomes (Sevignani et al., 2007; Rossi et al., 2008). These findings corroborate our data that miRNAs associated with cancer/cardiovascular disease are unevenly distributed in the human chromosomes and certain chromosomes have relatively higher disease-associated miRNA counts like chromosome 19, 14, X, and 1. In other-words, certain chromosomes are most likely controlling major cellular processes including the disease onset and progression in different species through miRNA. Further, we see that almost 46% and 24% of total intergenic miRNAs are associated with cancer and cardiovascular diseases respectively whereas about 17% and 6% of total intronic miRNAs are involved in cancer and cardiovascular diseases. Although the number of intronic miRNAs is greater than intergenic miRNAs in human, the latter group contributes larger for these two disease onset and progression. One possible reason behind this observation could be as follows. Since the clustered miRNAs are mostly involved in any particular cellular pathway including disease onset/progression etc. (Willimott and Wagner, 2012; Godnic et al., 2013) and their origin is mostly from intergenic portion (Chien et al., 2011), and so we get intergenic miRNAs sub-type is predominantly associated with cancer and cardiovascular diseases. Since the intronic miRNAs are mostly by-product of transcription/post-transcription processing of corresponding host protein coding genes, one can look into detailed expression profile of the coding genes which hosts those disease-associated intronic/exonic/others miRNAs for better understanding of onset and progression of such diseases. Furthermore, regulation of biogenesis of miRNAs especially intergenic miRNAs will help us to understand some of the molecular mechanisms of pathogenesis of these two diseases.

## Materials and methods

### Data collection

#### Details of chromosomal information

We collected the details of each chromosome like length of the chromosome, number of coding genes and non-coding genes in that chromosome for 20 different species (Figure 1) in metazoans from the karyotype data using Ensembl genome browser (release 74- December 2013), jointly maintained by European Bioinformatics Institute (EBI) and Welcome Trust Sanger Institute (WTSI), UK (Flicek et al., 2012). We used the following Genome assemblies for each species in this work- ASM23792v1 [*S. mansoni*], BDGP5 (GCA_000001215.1) [*D. melanogaster*], WBcel235 (GCA_000002985.3) [*C. elegans*], KH (GCA_000224145.1) [*C. intestinalis*], AnoCar2.0 (GCA_000090745.1) [*A. carolinensis*], Galgal4 (GCA_000002315.2) [*G. gallus*], taeGut3.2.4 [*T. guttata*], Zv9 (GCA_000002035.2) [*D. rerio*], MEDAKA1 [*O. latipes*], TETRAODON8 [*T. nigroviridis*], Sscrofa10.2 (GCA_000003025.4) [*S. scrofa*], OANA5 (GCF_000002275.2) [*O. anatinus*], BROADO5 (GCF_000002295.2) [*M. domestica*], GRCm38 (GCA_000001635.4) [*M. musculus*], Rnor_5.0 (GCA_000001895.3) [*R. norvegicus*], UMD3.1 (GCA_000003055.3) [*B. taurus*], MMUL_1 [*M. mulatta*], CHIMP2.1.4 (GCA_000001515.4) [*P. troglodytes*], gorGor3.1 (GCA_000151905.1) [*G. gorilla*], and GRCh37 (GCA_000001405.14) [*H. sapiens*].

#### Resource for miRNAs

miRBase (Griffiths-Jones et al., 2008) is a vastly used online registry of miRNAs of a wide range of species focusing on miRNA nomenclature, annotation and target prediction. We collected information about the chromosomal location of individual miRNA of a particular species from miRBase (Release 20: June 2013). We also retrieved the details of each miRNAs and categorized them into four groups according to their genomic location such as intergenic, intronic, exonic and others. We created tables with columns of chromosome number, chromosome length, number of coding/non-coding genes and number of miRNAs in respective chromosomes for each species. We downloaded the miRNAs associated with cancer and cardiovascular diseases from HMDD v1.0—a human miRNA disease database (Lu et al., 2008).

### Statistical analysis

We calculated Pearson product-moment correlation coefficient between column of miRNAs with the column of chromosome length, coding genes and non-coding genes for each species using Microsoft Office Excel 2003 software.

## Conflict of interest statement

The authors declare that the research was conducted in the absence of any commercial or financial relationships that could be construed as a potential conflict of interest.
